# Dietary Amylose/Amylopectin Ratio Modulates Cecal Microbiota and Metabolites in Weaned Goats

**DOI:** 10.3389/fnut.2021.774766

**Published:** 2021-11-23

**Authors:** Kefyalew Gebeyew, Kai Chen, Teketay Wassie, Md. Abul Kalam Azad, Jianhua He, Weimin Jiang, Wu Song, Zhixiong He, Zhiliang Tan

**Affiliations:** ^1^CAS Key Laboratory for Agro-Ecological Processes in Subtropical Region, National Engineering Laboratory for Pollution Control and Waste Utilization in Livestock and Poultry Production, Hunan Provincial Key Laboratory of Animal Nutritional Physiology and Metabolic Processes, Institute of Subtropical Agriculture, The Chinese Academy of Sciences, Changsha, China; ^2^University of Chinese Academy of Sciences, Beijing, China; ^3^College of Animal Science and Technology, Hunan Agricultural University, Changsha, China; ^4^Herbivore Nutrition Department, Hunan Institute of Animal and Veterinary Science, Changsha, China

**Keywords:** amylose/amylopectin ratio, cytokines, goats, metabolite, microbiota

## Abstract

Increasing the ratio of amylose in the diet can increase the quantity of starch that flows to the large intestine for microbial fermentation. This leads to the alteration of microbiota and metabolite of the hindgut, where the underlying mechanism is not clearly understood. The present study used a combination of 16S amplicon sequencing technology and metabolomics technique to reveal the effects of increasing ratios of amylose/amylopectin on cecal mucosa- and digesta-associated microbiota and their metabolites in young goats. Twenty-seven Xiangdong black female goats with average body weights (9.00 ± 1.12 kg) were used in this study. The goats were randomly allocated to one of the three diets containing starch with 0% amylose corn (T1), 50% high amylose corn (T2), and 100% high amylose corn (T3) for 35 days. Results showed that cecal valerate concentration was higher (*P* < 0.05) in the T2 group than those in the T1 and T3 groups. The levels of tumor necrosis factor-α (TNF-α) and interleukin (IL)-6 were decreased (*P* < 0.05) in cecal tissue while IL-10 was increased (*P* < 0.05) in the T2 group when compared with T1 or T3 groups. At the phylum level, the proportion of mucosa-associated Spirochaetes was increased (*P* < 0.05), while Proteobacteria was deceased by feeding high amylose ratios (*P* < 0.05). The abundance of Verrucomicrobia was decreased (*P* < 0.05) in the T3 group compared with the T1 and T2 groups. The abundance of digesta-associated Firmicutes was increased (*P* < 0.05) while Verrucomicrobia and Tenericutes were deceased (*P* < 0.05) with the increment of amylose/amylopectin ratios. The LEfSe analysis showed that a diet with 50% high amylose enriched the abundance of beneficial bacteria such as *Faecalibacterium* and *Lactobacillus* in the digesta and *Akkermansia* in the mucosa compared with the T1 diet. The metabolomics results revealed that feeding a diet containing 50% high amylose decreased the concentration of fatty acyls-related metabolites, including dodecanedioic acid, heptadecanoic acid, and stearidonic acid ethyl ester compared with the T1 diet. The results suggested that a diet consisting of 50% high amylose could maintain a better cecal microbiota composition and host immune function.

## Introduction

Ruminants possess a better potential to degrade large quantities of starch, which has a different degree of resistance to degradation. Several factors determine the rate and extent of starch digestion in the rumen, such as crystallinity, a ratio of amylose/amylopectin, particle size, and cooking approach (1, 2). Amylose and amylopectin are the main components of starch and can determine the nutritional and biochemical properties of starch (1). Amylopectin is a highly branched glucose polymer with α−1, 6-glucoside bonds and has a higher rate of digestion than amylose (2). Unlike amylopectin, amylose is characterized by a linear glucose polymer and not easily hydrolyzed due to the formation of granules by linear amylose molecules that resist degradation by mammalian enzymes (3).

Feeding a high rumen degradable starch increased the risk of intestinal inflammation in the hindgut of dairy goats, as evidenced by an increase in the expression of interleukin-1β and secretory immunoglobulin A (SIgA), and accumulate short-chain fatty acids (SCFAs), and disturbance of microbial function (4). Developing efficient feeding approaches for ruminant animals needs the maintenance of optimal rumen and hindgut fermentation in the modern feeding system. To address this issue, the inclusion of an optimum ratio of amylose/amylopectin in the diets may be an effective approach to take advantage of shifting starch degradation sites from the forestomach to the intestine, including improving energetic efficiency and the hepatic glucose supply and reducing methane production (5, 6). To optimize diet formulation, we need to understand the changes induced by feeding of starch-containing different ratios of amylose/amylopectin on the hindgut fermentation profiles and metabolites. In addition to that, many studies have investigated how starch type effects on the animal performance. However, the ratios of amylose to amylopectin are very close. This study used high amylose corn to enlarge the gap of amylose to amylopectin ratio between low and high treatment. In this case, it may be more suitable to see the effect of the ratio of amylose to amylopectin on animals.

The quantity of starch reaching the hindgut region affects the intestinal microbial structure and end-products of microbial fermentation. Several studies have pointed out that starch with a high amylose proportion modulates the intestinal microbiota and metabolites, leads to shifts in SCFAs profiles, microbial composition, and immune status (7, 8). Feeding a diet containing high amylose starch increases the abundance of commensals bacterial, such as *Lactobacilli* and *Faecalibacterium*, and reduces pathogenic microbes, including *Salmonella*, in the hindgut region (9). These changes might contribute to positive outcomes in the host health and productivity either directly or indirectly. However, an ideal dietary amylose concentration should be determined to achieve better efficiency and intestinal health under a certain animal growth stage or physiological conditions. It has been reported that the growth rate and butyrate production are lower by feeding a starch with a 63% amylose proportion (10), which is not an appreciated outcome. Supplementing different ratios of amylose/amylopectin to weaned piglets experiencing feed transition or exposed with *E.coli* lipopolysaccharide have given distinct outcomes (11), suggesting the inclusion of optimal ratio is critical to gain better efficiency.

The cecum is one of the primary fermentation regions of the hindgut of goats. It has been widely accepted that cecal microbes can metabolize the undigested substrate into various compounds, including SCFAs, organic acids, lipids, and phenols (8). Metabolites from those compounds have shown a wide range of biological functions such as serving as signaling molecules, source of energy, and becoming integrated into other molecules (12, 13), which are eliciting systemic effects. A study using rats shows that the levels of metabolites related to lipids metabolism, such as heptadecanoic acid, are reduced by feeding high-amylose maize-resistant starch type 2 (7). This metabolite has several biological and nutritional roles and serves as a biomarker of various diseases (14). Taken together, a holistic dietary intervention study that has comprehensively assessed the effects of different ratios of amylose/amylopectin on the hindgut microbiome and metabolome is required. To address this, the metabolomics approach can identify several metabolites in complex tissue or biofuel samples, which provides key insight into the interaction between the intestinal microbiome and host metabolism and uncovering possible metabolic biomarkers of intestinal health (12). Recently, an untargeted metabolomics approach was used to assess metabolites alteration in the colon of mice fed xylitol (15), which gives insight into the role of metabolomics techniques in nutritional studies. Thus, a combination of molecular approaches was used to gain a clear picture of the effects of increasing ratios of dietary amylose to amylopectin on cecum mucosa-and digesta-associated microbiota and their metabolites in weaned goats.

## Materials and Methods

### Animals, Diets, and Experimental Design

Twenty-seven Xiangdong black female goats with average body weights (BWs) of 9.00 ± 1.12 kg and aged about 2 months were used in this study. The weaned goats were randomly allocated into one of three diets containing starch with 0% amylose corn (T1), 50% high amylose corn (T2), and 100% high amylose corn (T3). The diets were prepared according to the feeding standard of Chinese goats (16). Details about the ingredients and nutrient composition of the experimental diets are presented in Table 1. Ratios of amylose/amylopectin were formulated by using different corn variety (Hainan Shanliang Technology Co., Ltd., Haikou, Hainan). The feeding trial period consisted of 35 days for the actual experiment and 14 days for adaptation periods. During the trial period, goats were received concentrate and alfalfa twice daily at 08:00 and 16:00 h. All goats had *ad libitum* access to water, concentrate, and alfalfa during the experimental periods.

**Table 1:** Ingredients and chemical composition (% of DM) of the treatment diets.

**Item**	**Treatment^a^**		
	**T1**	**T2**	**T3**
**Ingredient, % of DM**			
Normal corn	83.0	41.5	0.0
High amylose-corn^b^	0.0	41.5	83.0
Soybean meal	12.0	12.0	12.0
CaHPO_4_·2H_2_O	1.5	1.5	1.5
CaCO_3_	0.7	0.7	0.7
Salt	0.8	0.8	0.8
Premix^c^	2.0	2.0	2.0
**Chemical composition, % of DM**			
DM	87.6	87.2	87.4
DE (KJ/Kg)	15.5	16.0	16.2
CP	11.1	12.0	12.0
Total starch	55.1	50.3	50.3
Amylose/Total starch	20.83	37.94	64.70
Amylopectin/Total starch	79.17	62.06	35.30
Amylose/amylopectin	0.26	0.61	1.81
Ca (%)	0.7	0.7	0.8
TP (%)	0.3	0.3	0.3

*Nutritional composition of alfalfa: DM 95.9%, CP 14.5%, NDF 37.2%, ADF 28.1%*.

a *T1 (normal corn 100%, high amylose corn 0%); T2 (normal corn 50%, high amylose corn 50%); T3 (normal corn 0%, high amylose corn 100%). DM, Dry matter; DE, Digestive energy; CP, Crude protein; TP, total phosphorus*.

b *High amylose-corn was provided by Hainan Shanliang Technology Co., Ltd. (Haikou, Hainan)*.

c *The premix provided the following per kilogram of the diet: MnSO_4_·H_2_O 15.33 g, FeSO_4_·7H_2_O 30 g, CuSO_4_·5H_2_O 25.33 g, ZnSO_4_·H_2_O 15.33 g, iodine 0.667 g, selenium 0.67 g, cobalt 0.67 g, Vitamin A 32,500 IU, Vitamin D_3_ 10,000 IU, Vitamin E 80 IU, Vitamin K_3_ 10 mg, Vitamin B1 10 mg, Vitamin B_2_ 25 mg, Vitamin B6 8 mg, Vitamin B_12_ 0.075 mg, biotin 0.600 mg, folic acid 5 mg, nicotinamide 100 mg, pantothenic acid 50 mg*.

### Sample Collection

On day 35, all goats were banned but provided free access to water for 12 h and then slaughtered by a registered veterinarian. The cecum mucosa was collected quickly after slaughter and rinsed three times with cold phosphate buffer saline. The cecum tissue was divided into two portions. The first portion was cut into smaller pieces, then quickly flash-frozen in liquid nitrogen for cytokines measurements. The second portion was scraped from the underlying tissue using a clean glass slide, quickly moved into liquid nitrogen and kept at −80°C until DNA extraction. Meanwhile, the cecum digesta was divided into two portions. About 10 g of each cecum digesta sample was mixed thoroughly with deionized water. The mixtures were quickly centrifuged at 3,000 × *g*, and the supernatants were stored at −80°C until SCFAs analysis. The second portion was stored at −80°C until DNA extraction.

### Measurement of Cecum SCFA

Frozen cecum digesta were thawed on ice and centrifuged at 20,000 × *g* for 10 min. About 1.5 mL of the supernatants were transferred into 2 mL of plastic tubes containing 0.15 mL of 25% (wt/vol) metaphosphoric acid. The mixtures were strongly hand-shaken and kept at −20°C for overnight for analysis. Afterward, re-centrifuging at 15,000 × *g* for 10 min, 1 mL aliquots were collected into an EP tube passed through a 0.22-μm filter membrane. The concentrations of SCFA were assayed by gas chromatography (GC, Agilent 7890A, and Agilent Inc., Santa Clara, CA) following the procedure previously described by Wange et al. (17). The concentrations of SCFA were presented as μmol/g fresh weight of digesta.

### Measurement of Cecum Cytokines Levels

About 100 mg of the cecum mucosa samples were homogenized with ice-cold deionized water and centrifuged at 3,000 × *g* for 15 min at 4°C, and then the upper liquids were collected to detect the levels of cytokines. The levels of interleukin (IL)-2, IL-6, IL-10, tumor necrosis factor-α (TNF-α), and interferon-β (INF-β) were detected using the Goat enzyme-linked immunosorbent assay (ELISA) kits according to the manufacturer's directions (Jiangsu Yutong Biological Technology Co., Ltd., China) (18). The levels of cytokines were expressed as pg/mg protein. The concentrations of protein in the cecal mucosa were measured using the bicinchoninic acid procedure (BCA Protein Assay Kit; Beyotime Biotech Inc, Shanghai, China) using bovine serum albumin as the standard.

### Genomic DNA Extraction

Eight samples from each treatment were randomly selected for the 16S rRNA gene sequencing. Genomic DNA was isolated from tissue and digesta samples using the modified Power Soil^®^ DNA Isolation Kit (MoBio Laboratories, Inc., Carlsbad, CA, USA) according to the manufacturer's protocol. Briefly, genomic DNA was extracted using 250 mg of starting materials, a dry bead tube, and a 750 μL of bead solution. Mechanical cell lysis (bead-beating) was performed at 50 Hz for 5 min using the TissueLyser LT™ (Qiagen, FRITSCH GmbH, Idar-Oberstein, Germany). The lysate was incubated at 65°C for 10 min, and then the supernatant was collected for further process. The extracted DNA was eluted in 50 μl of elution buffer and stored at −80°C until subsequent steps. The quality and quantity of extracted DNA were assessed using a ND-1000 spectrophotometer (NanoDrop Technologies Inc., Wilmington, United States).

### PCR Amplification and 16S rRNA Gene Sequencing

The amplicon library was constructed by PCR amplification of the V3–V4 hypervariable region of the 16S rRNA gene using the 338F (5'-ACTCCTACGGGAGGCAGCAG-3') and 806R (5'-GGACTACHVGGGTWTCTAAT-3') primers with barcodes (19). The PCR reactions were performed at 98°C denaturation for 30 s, 32 cycles of 10 s at 98°C, 30 s of annealing at 54°C and 45 s of elongation at 72°C; and last extension at 72°C for 10 min. The PCR reaction mixture consisted of 25 ng of template DNA, 2.5 μL of each primer, 12.5 μL PCR Premix, and PCR-grade water to 25 μL (20). The PCR products were run on 2% agarose gel electrophoresis and then excised, and further purified by AMPure XT beads (Beckman Coulter Genomics, Danvers, MA, USA). The purified PCR products were quantified by Qubit (Invitrogen, Waltham, MA, USA). Amplicon pools were used for sequencing, and the size and quantity of the amplicon library were determined using an Agilent 2100 Bioanalyzer (Agilent, Santa Clara, CA, USA) and an Illumina Library Quantification Kit (Kapa Biosciences, Woburn, MA, USA). The sequencing library was constructed using a TruSeq^®^DNA PCR-free library preparation kit (Illumina, San Diego, CA, USA) following the manufacturer's guidelines. After the library quality control was finished, the libraries were sequenced by BMK Cloud (Biomarker Technologies Co., Ltd., Beijing, China) using the HiSeq 2500 platform (2 × 250 paired ends; Illumina Technologies Co. Ltd, San Diego, CA, USA).

### Bioinformatics Analysis

According to their unique barcodes, truncated paired-end reads were assigned to appropriate samples and combined using FLASH software (21). Quality filtering of the raw tags was achieved using fqtrim (v0.94) as described by Zhang et al. (22). Chimeric sequences were cleaned up using Vsearch software (v2.3.4) (23). Sequences with similarity ≥ 97% were clustered into the same operational taxonomic unit (OTUs) using USEARCH (Version 10.0) (24). Taxonomy annotation of the OTUs was conducted based on the Ribosomal Database Project (RDP) classifier using the SILVA database (release132) with a confidence threshold of 80% (25). The population evenness (Shannon index) and richness (Chao1) were estimated using QIIME2 (V1.8.0) (26). Principal coordinate analysis (PCoA) was performed using UniFrac distance metrics. Analysis of similarity (ANOSIM) was used to test the statistical differences among the groups. Linear discriminant analysis (LDA) effect size (LEfSe) was performed to reveal the difference in the bacterial communities among the treatments using the non-parametric factorial Kruskal-Wallis test with an alpha value of 0.05 and LDA score of 2.5 (18). The raw reads were deposited at NCBI under BioProject accession ID: PRJNA759377.

### Non-targeted Metabolomics Analysis

The six samples from each treatment of the cecal tissues were randomly selected for the metabolomics analysis. The cecum tissue was thawed on ice, and metabolites were extracted with 50% methanol buffer (27). Briefly, 20 μL of the sample were extracted with 120 μL of precooled 50% methanol, vortexed for 1 min, and incubated at room temperature (24 ± 2°C) for 10 min. The samples were stored at −80°C prior to the LC-MS analysis. In addition, pooled QC samples were prepared by combining 10 μL of each extraction mixture stored at −80°C prior to the LC-MS analysis. All samples were acquired by using the LC-MS system according to the direction of the instrument. All chromatographic separations were performed following the protocol as previously described in the study of (28) using an ultra-performance liquid chromatography system (SCIEX, UK). A high-resolution tandem mass spectrometer TripleTOF5600plus (SCIEX) was used to identify metabolites eluted from the column according to the procedure previously described by Xiang et al. (15). The obtained MS data pretreatments, including peak picking and grouping, correction of retention time (RT), grouping of second peak, and annotation of isotopes and adducts, were performed using XCMS software 3.2.0 (UC, Berkeley, CA, USA). Student's *t*-tests were employed to detect differences in metabolites concentrations between the groups. Supervised PLS-DA was performed using MetaX to discriminate the different variables between groups (29). Differentially accumulated metabolites (DAMs) in content were defined as having variable importance in the project (15) ≥ 1 and a fold change of ≥ 2 or ≤ 0.5.

### Statistical Analysis

Statistical analyses of the experimental data were carried out using the SPSS version 23 (SPSS Inc., Chicago, IL, USA) and Origin Pro 2020b software. The SCFA and intestinal immune parameters were subjected to a one-way analysis of variance (ANOVA) procedure after checked for normality and homogeneity of variance. The difference between the three groups was evaluated using Tukey *post-hoc* tests, and differences were considered statistically significant at *P* < 0.05, and trends were recognized 0.05 < *P* < 0.1. The effect of diets on the alpha diversity and relative abundance of detected bacterial groups was evaluated using the non-parametric Kruskal-Wallis test in Origin Pro 2020b software. Means ± standard errors of the mean (SEMs) were used to present the results. The correlations between bacteria at the genus level and SCFAs were analyzed by Spearman's rank correlation test using the OmicStudio tools at https://www.omicstudio.cn/tool (LC-Bio Technology Co., Ltd., Hangzhou, China).

## Results

### Microbial SCFAs Concentrations in the Cecum Digesta

**Figure 1:**
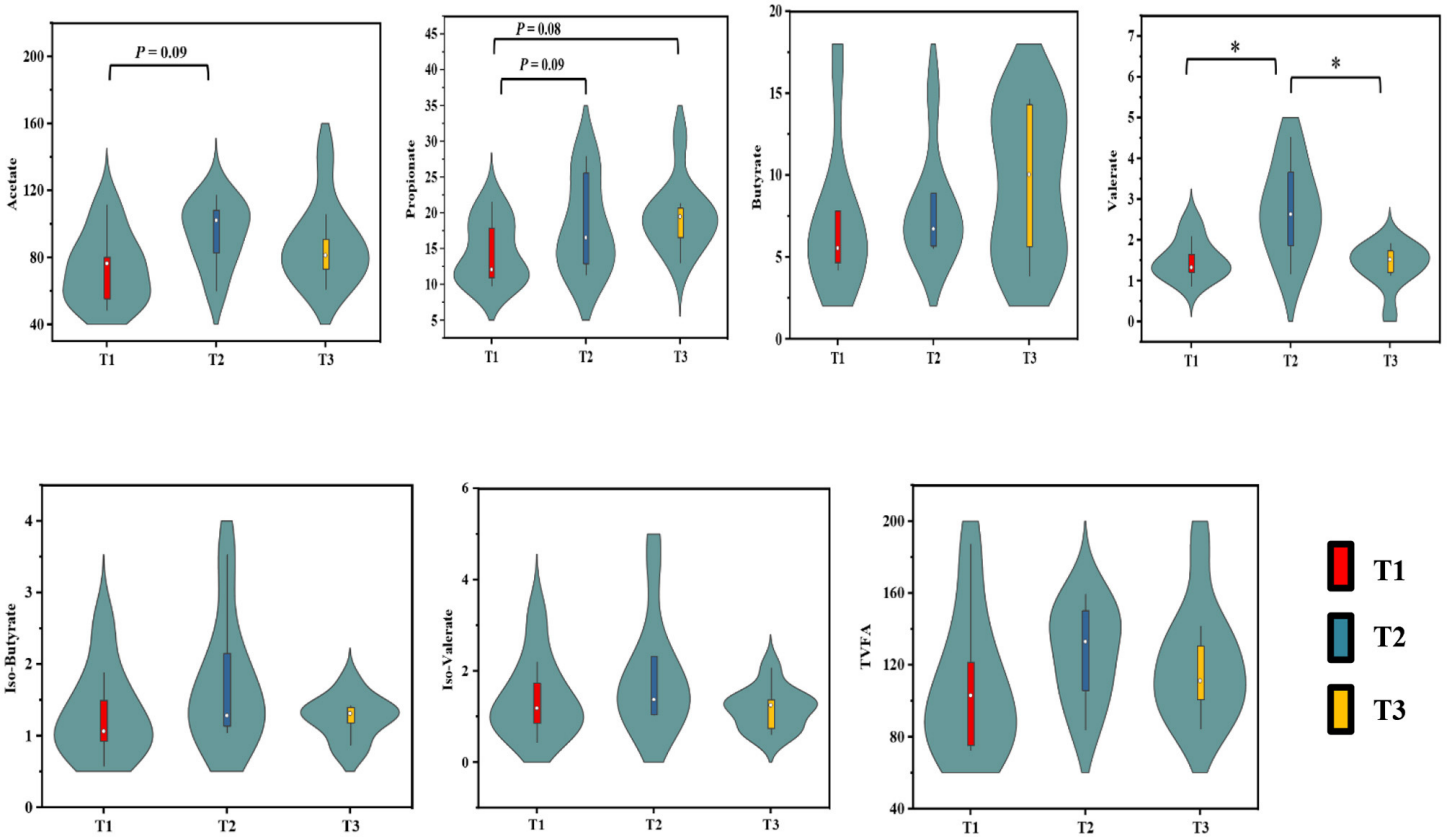
Effects of dietary amylose/amylopectin ratio on cecal SCFAs concentrations (μmol/g). On each side of the gray line is a kernel density estimation to illustrate the distribution of shape of the data in a group. Wider and skinnier sections of the violin plot represent a higher and lower probability that members of the samples will take on the given value. The white dot represents the median and the thick gray bar in the center represents the interquartile range. *Significantly different means (*P* < 0.05). T1 (normal corn 100%, high amylose corn 0%); T2 (normal corn 50%, high amylose corn 50%); T3 (normal corn 0%, high amylose corn 100%).

The concentrations of SCFAs in the cecum digesta of weaned goats in response to feeding different ratios of amylose/amylopectin are presented in Figure 1. The concentrations of acetate in the T2 group (*P* = 0.09) and propionate in the T2 (*P* = 0.09) and T3 (*P* = 0.08) groups were tended to increase when compared with the T1 group. The valerate concentration was higher in the T2 than that in the T1 and T3 groups (*P* < 0.05). However, the concentrations of butyrate, iso-butyrate and iso-valerate were unaffected (*P* > 0.05) when the amylose/amylopectin ratio increased.

### Cytokines Concentrations in the Cecum Mucosa

**Figure 2:**
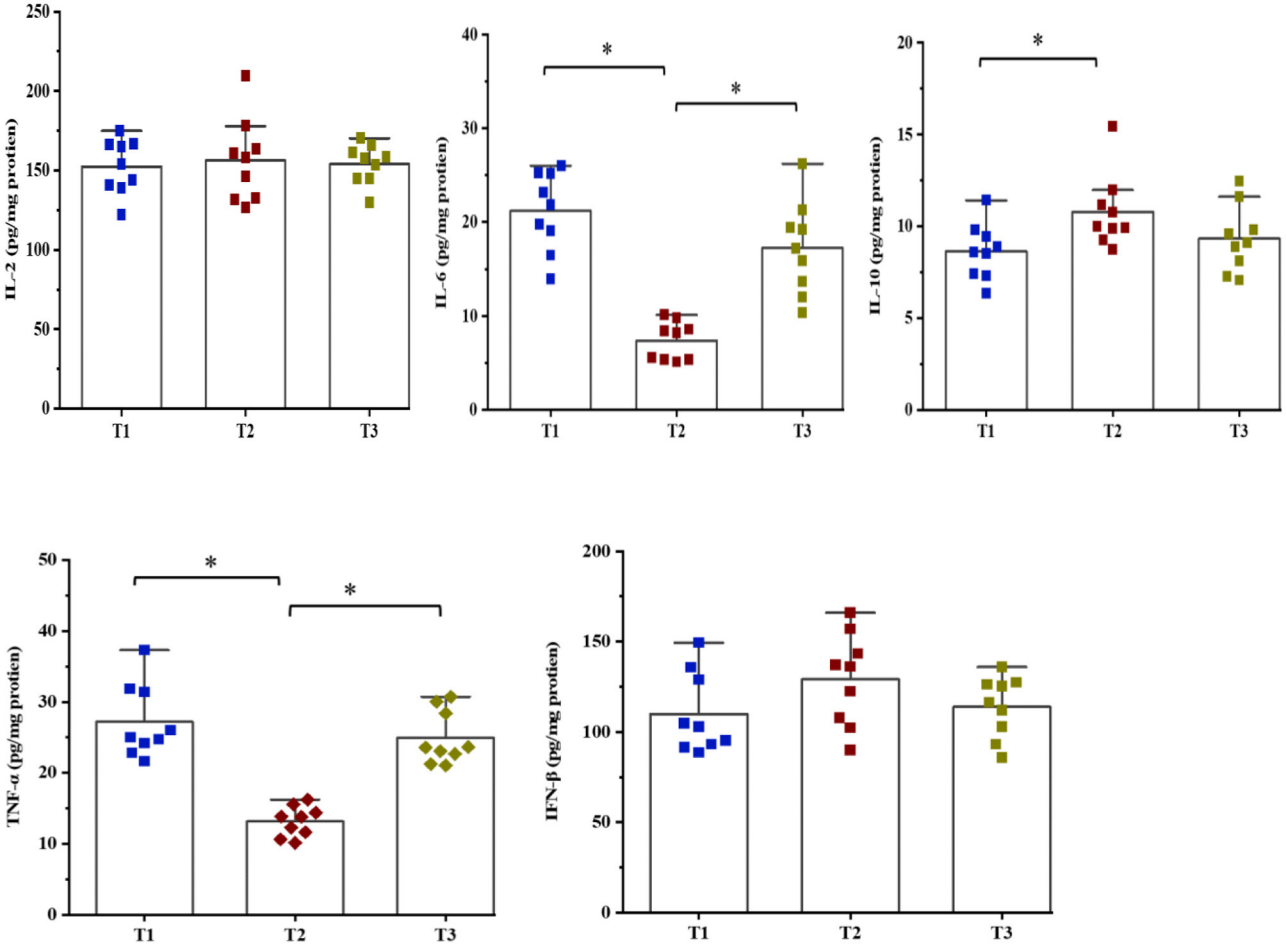
Effects of dietary amylose/amylopectin ratio on cecal cytokines concentrations (pg/mg protein). Values are expressed as means ± SEM indicated by vertical bars. *Significantly different means (*P* < 0.05). T1 (normal corn 100%, high amylose corn 0%); T2 (normal corn 50%, high amylose corn 50%); T3 (normal corn 0%, high amylose corn 100%).

The concentrations of cytokines were determined to evaluate the effects of amylose/amylopectin ratios on immune response in weaned goats (Figure 2). The concentrations of IL-6 and TNF-α were decreased (*P* < 0.05) while IL-10 was increased (*P* < 0.05) in the T2 groups compared with the T1 or T3 groups. No significant differences (*P* > 0.05) in the concentrations of IL-2 and IFN-β were found among the three groups.

### The Mucosa-Associated Microbiome

**Figure 3:**
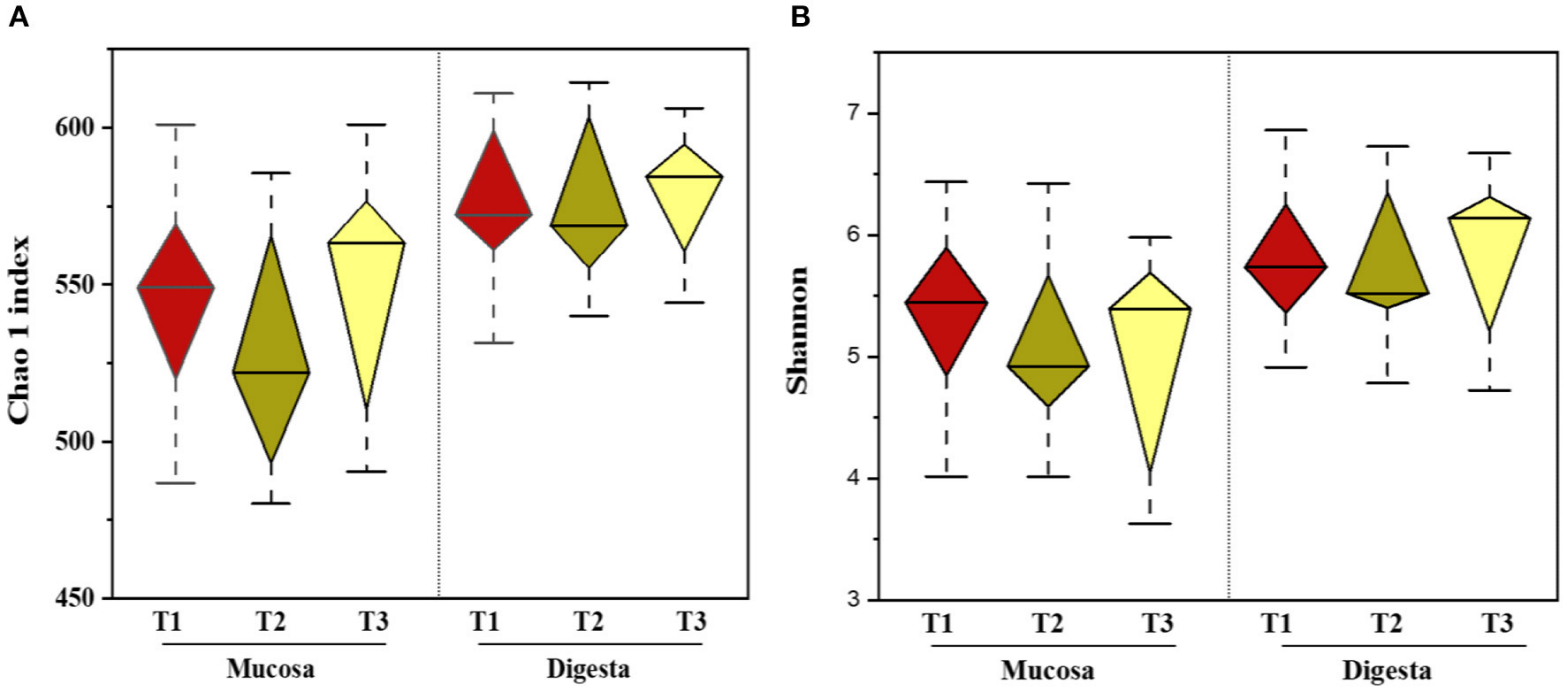
Effects of dietary amylose/amylopectin ratio on alpha-diversity indices in the cecal mucosa and digesta **(A)** Chao 1 Index. **(B)** Shannon index. Diamond plot of richness and evenness diversity values showed that the microbiota structure of each group. Whiskers represent minimum and maximum value. Bottom and top of the box are the first and the third quartile. The median is shown as a band inside the box. T1 (normal corn 100%, high amylose corn 0%); T2 (normal corn 50%, high amylose corn 50%); T3 (normal corn 0%, high amylose corn 100%).

**Figure 4:**
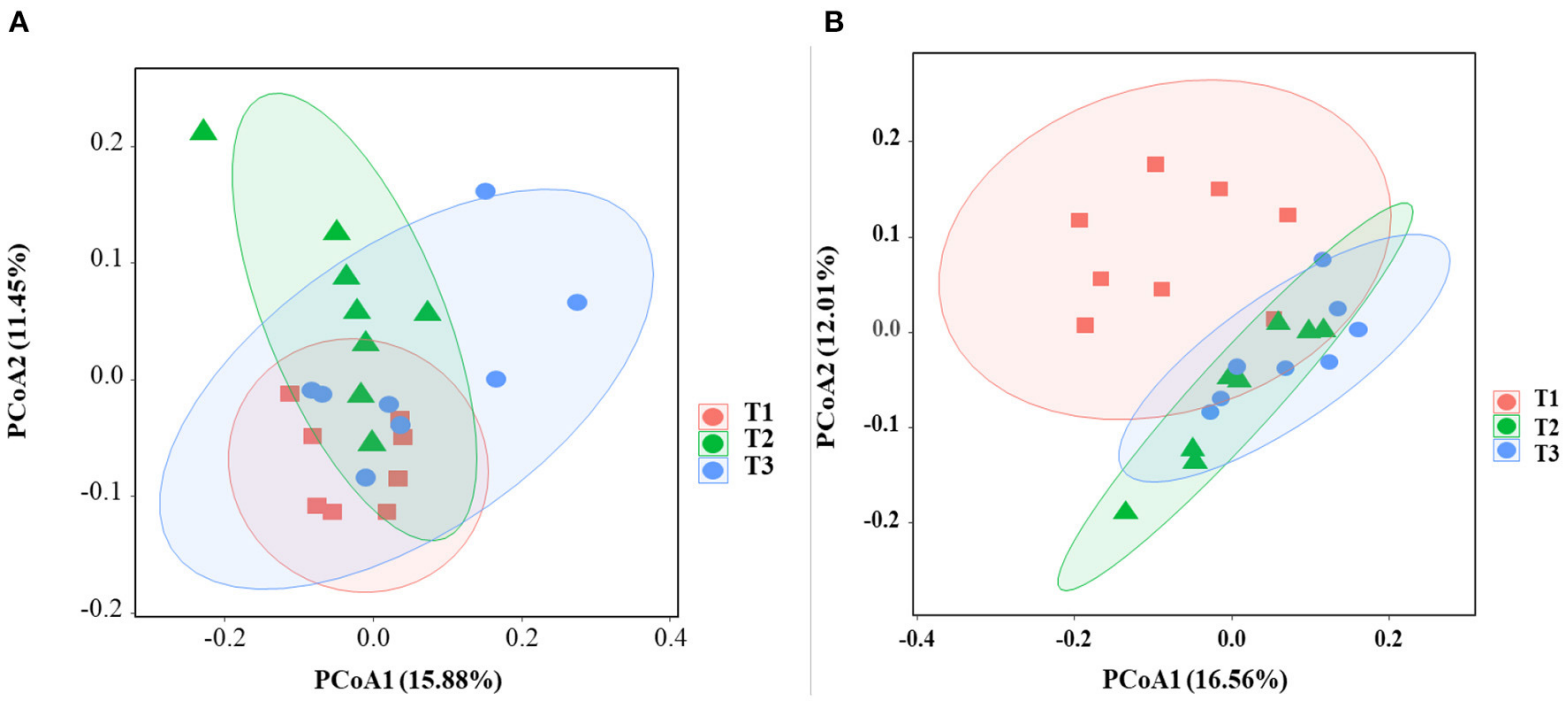
Effects of dietary amylose/amylopectin ratio on beta-diversity in the cecal mucosa **(A)** and digesta **(B)**. The PCoA plot was generated using unweighted UniFrac-based. T1 (normal corn 100%, high amylose corn 0%); T2 (normal corn 50%, high amylose corn 50%); T3 (normal corn 0%, high amylose corn 100%).

**Figure 5:**
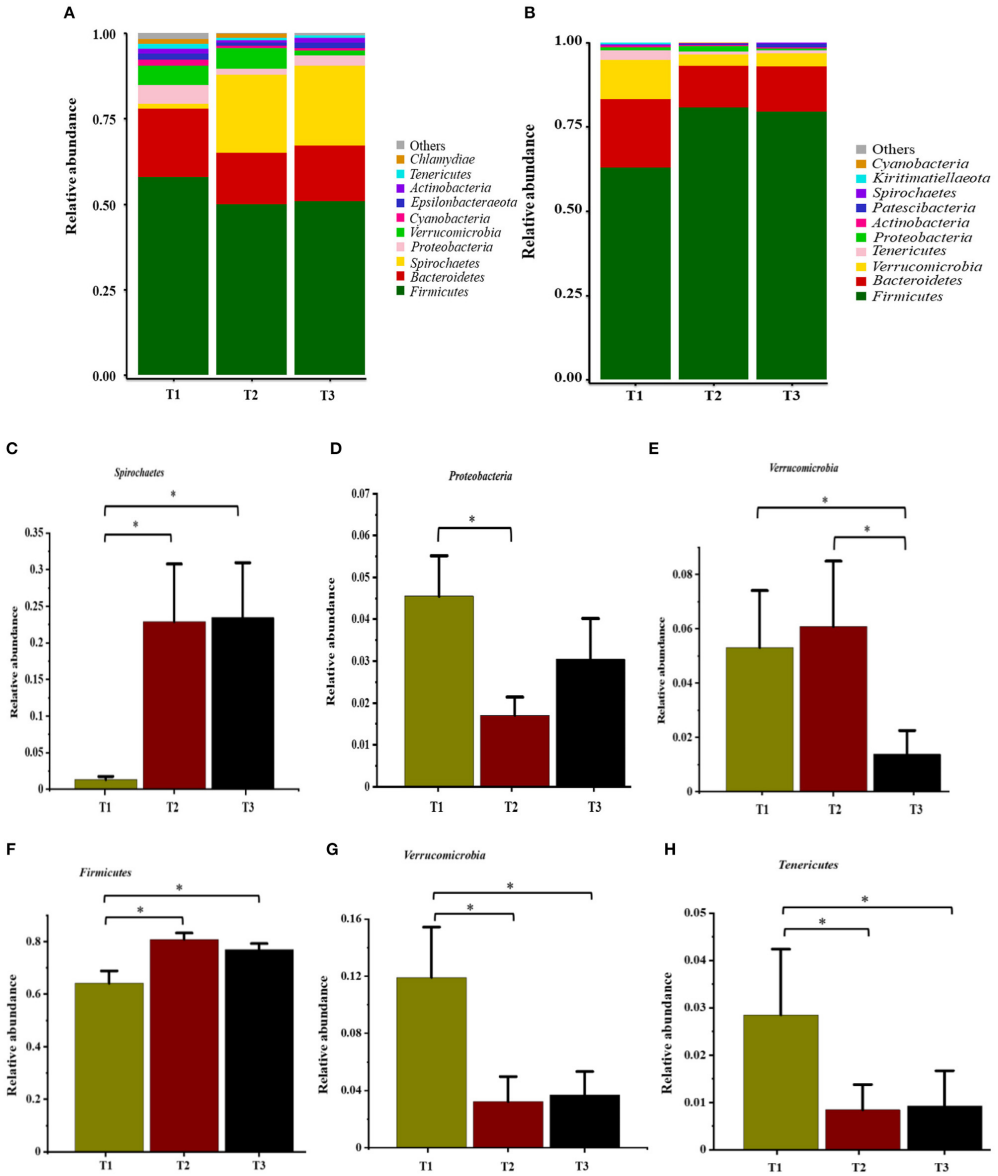
Relative abundance of bacteria at the phylum level in the cecal mucosa **(A)** and digesta **(B)**. Comparison of relative abundances at phylum levels (**C–E**, mucosa) and (**F–H**, digesta) were analyzed by the Kruskal-Wallis rank-sum test. Values are expressed as means ± SEM indicated by vertical bars. *Significantly different means (*P* < 0.05). T1 (normal corn 100%, high amylose corn 0%); T2 (normal corn 50%, high amylose corn 50%); T3 (normal corn 0%, high amylose corn 100%).

**Figure 6:**
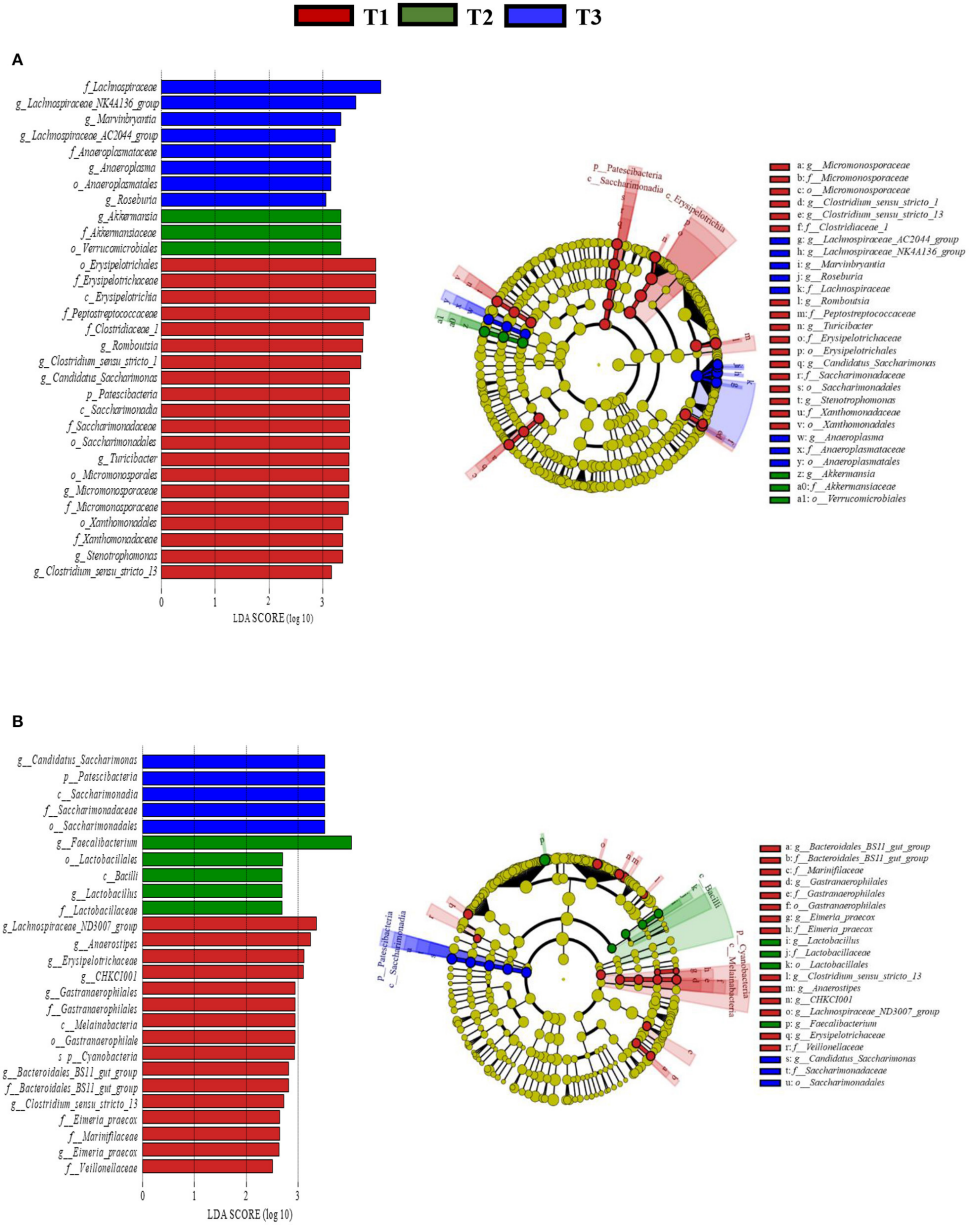
Linear discriminant analysis (LDA) effect size (LEfSe) analysis identified the most differentially abundant from phylum to genus in the cecal mucosa **(A)** and digesta **(B)** between T1 (red), T2 (Green), and T3 (Blue) groups. The genus with linear discriminant analysis values higher than 2.5 is displayed. The length of the bar column represents the LDA score. The cladogram, circles radiating from inner side to outer side represents the differences in the relative abundance of taxa from phylum to genus level between the three groups. T1 (normal corn 100%, high amylose corn 0%); T2 (normal corn 50%, high amylose corn 50%); T3 (normal corn 0%, high amylose corn 100%).

In total, 1,791,154 valid reads were retrieved from cecal mucosa samples with an average of 66,339 sequences per sample after quality trimming and chimera checking. The minimum and maximum nucleotides lengths were 410 and 425, respectively, and the three groups shared 770 OTUs. The pattern of the rarefaction curve confirmed that the sequencing data coverage was adequate to describe the mucosa-associated bacterial composition in the cecum of goats used in the present study (Supplementary Figure 1). The Chao1 and Shannon indices were used to assess the alpha diversity of mucosa-associated microbial profiles. No significant differences (*P* > 0.05) in bacterial community richness and diversity were observed among the three groups (Figure 3A). The PCoA revealed that both the T2 and T3 groups were formed separate clusters from the T1 group (ANOSIM-R = 0.1093, *P* < 0.004), while the T2 and T3 groups were clustered together (ANOSIM-R = 0.0904, *P* = 0.064) (Figure 4A), suggesting that the communities share most OTUs. The relative abundances of mucosa-associated microbiota at the phylum level are shown in Figure 5A. There were 10 dominants of bacterial phyla identified with a mean relative abundance of ≥ 1%. Firmicutes (52.8 ± 5.9%), Bacteroidetes (16.9 ± 2.4%), and Spirochaetes (15.8 ± 5.2%) were the most dominant phyla and were represented with an average of more than 85% of the community. The relative abundance of Spirochaetes was significantly higher (*P* < 0.05) in the T2 and T3 groups than that in the T1 group, while no significant difference was found between the T2 and T3 groups (*P* > 0.05). In contrast, the relative abundance of Proteobacteria was lower (*P* < 0.05) in the T2 group compared with the T1 group. Verrucomicrobia had lower (*P* < 0.05) relative abundance in the T3 group compared with the T1 and T2 groups (Figures 5C–E). The LDA with LEfSe analysis was used to explore mucosa-associated microbiota differences from phylum to genus among the three groups (Figure 6A). The relative abundance of *Akkermansia* was enriched in the T2 group compared with the other two groups. The relative abundances of *Lachnospiraceae_NK4A136_group, Marvinbryantia, Roseburia*, and *Anaeroplasma* were enriched in the T3 group, while *Micromonosporaceae, Clostridium_sensu_stricto, Romboutsia, Turicibacter, Candidatus_Saccharimonas*, and *Stenotrophomonas* were enriched in the T1 group.

### The Digesta-Associated Microbiome

Overall, 2,105,981 sequences were generated from cecal-digesta samples with a mean of 68,867 sequences per sample. The minimum and maximum nucleotides lengths were 407 and 414, respectively, and a shared microbiota of 675 OTUs were found in all digesta samples. The pattern of rarefaction curves showed that the sequencing data coverage was satisfactory to describe the digesta-associated bacterial composition in the cecum of goats. No significant differences (*P* > 0.05) in Chao1 and Shannon indexes were observed among the three groups (Figure 3B). The PCoA showed that both the T2 and T3 groups clearly separated from the T1 group (ANOSIM-R = 0.3437, *P* < 0.001) according to the weighted UniFrac distance metric (Figure 4B), while the T2 and T3 groups were clustered together (ANOSIM-R = 0.1518, *P* = 0.061). The top 10 abundances of digesta-associated bacterial phyla with a mean relative abundance of ≥ 1% are shown in Figure 5B. Firmicutes (73.9 ± 3.1%), Bacteroidetes (15.2 ± 3.2%), and Verrucomicrobia (6.2 ± 2.3%) were the most dominant phyla and were represented with an average of more than 95% of the total population.

The relative abundance of Firmicutes was enriched (*P* < 0.05) in the T2 and T3 groups compared with the T1 group. Conversely, the relative abundances of Verrucomicrobia and Tenericutes were depleted (*P* < 0.05) in the T2 and T3 groups compared with the T1 group (Figures 5F–H). The LDA with LEfSe analysis revealed that *Bacteroidales_BS11_gut_group, Gastranaerophilales, Eimeria_praecox, Anaerostipes, Clostridium_sensu_stricto_13, CHKCI001, Lachnospiraceae_ND3007_group*, and *Erysipelotrichaceae* were enriched in the T1 group (Figure 6B). The relative abundances of *Lactobacillus and Faecalibacterium* from *Firmicutes* phylum were enriched in the T2 group, while *Candidatus_Saccharimonas* from *Patescibacteria* phylum was enriched in the T3 group.

### Correlation Analysis Between the Cecal Microbiome and SCFAs

**Figure 7:**
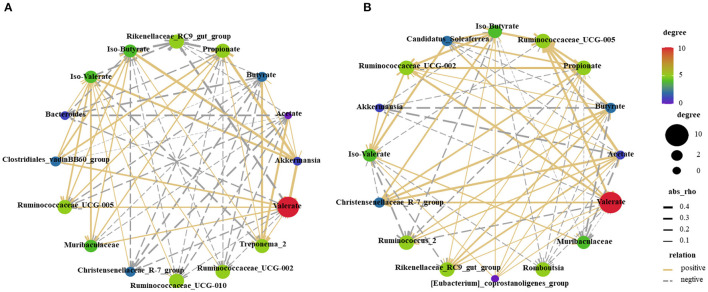
Correlations network analysis between SCFAs and the relative abundance of top 10 predominant bacteria at the genus level in the mucosa **(A)** and digesta **(B)** are presented. Silver lines, negative correlation (*P* ≤ 0.05); Golden lines, positive correlation (*P* ≤ 0.05).

The SCFAs and top 10 bacterial genera were used for Pearson's correlation analysis (Figure 7). The relative abundance of mucosa-associated *Akkermansia* showed a positive correlation (*P* < 0.05) with valerate, iso-valerate, propionate, and iso-butyrate. The relative abundance of digesta-associated *Ruminococcaceae_UCG-005* was positively correlated (*P* < 0.05) with propionate, iso-butyrate acetate, butyrate, and valeric. Meanwhile, the relative abundance of the digesta-associated *Christensenellaceae_R-7_group* was positively correlated (*P* < 0.05) with acetate and butyrate.

### Non-targeted Metabolome Profiles of the Cecal Tissue

**Figure 8:**
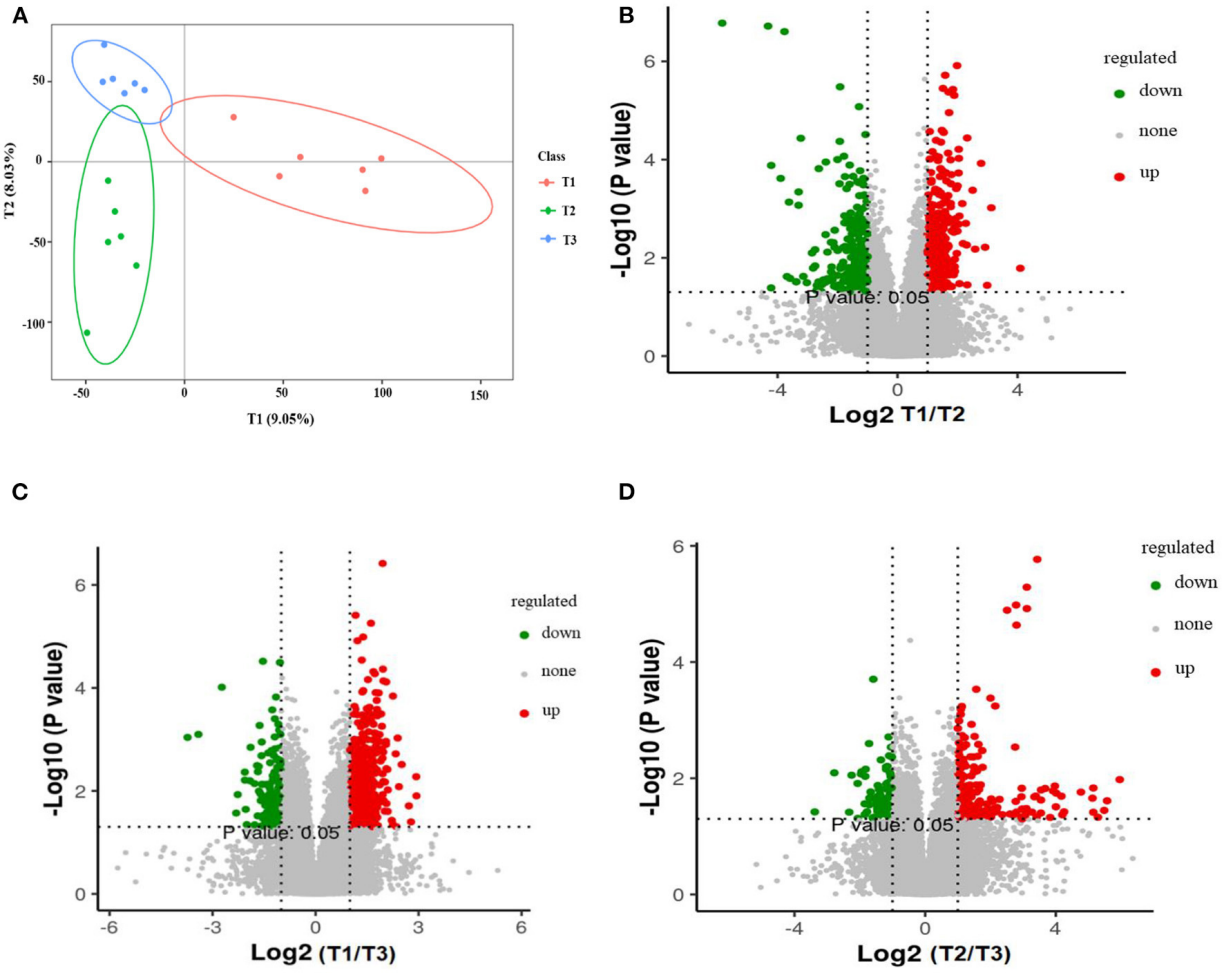
Partial least squares discriminant analysis (PLS-DA). The PLS-DA of microbial metabolites in cecal tissue of goats **(A)**. Volcano plot showing the differential metabolites (red dot: significantly up-produced metabolites, green dot: significantly down-produced metabolites, gray dot: the metabolites with no significant difference, *Q* < 0.05) **(B–D)**. T1 (normal corn 100%, high amylose corn 0%); T2 (normal corn 50%, high amylose corn 50%); T3 (normal corn 0%, high amylose corn 100%).

**Figure 9:**
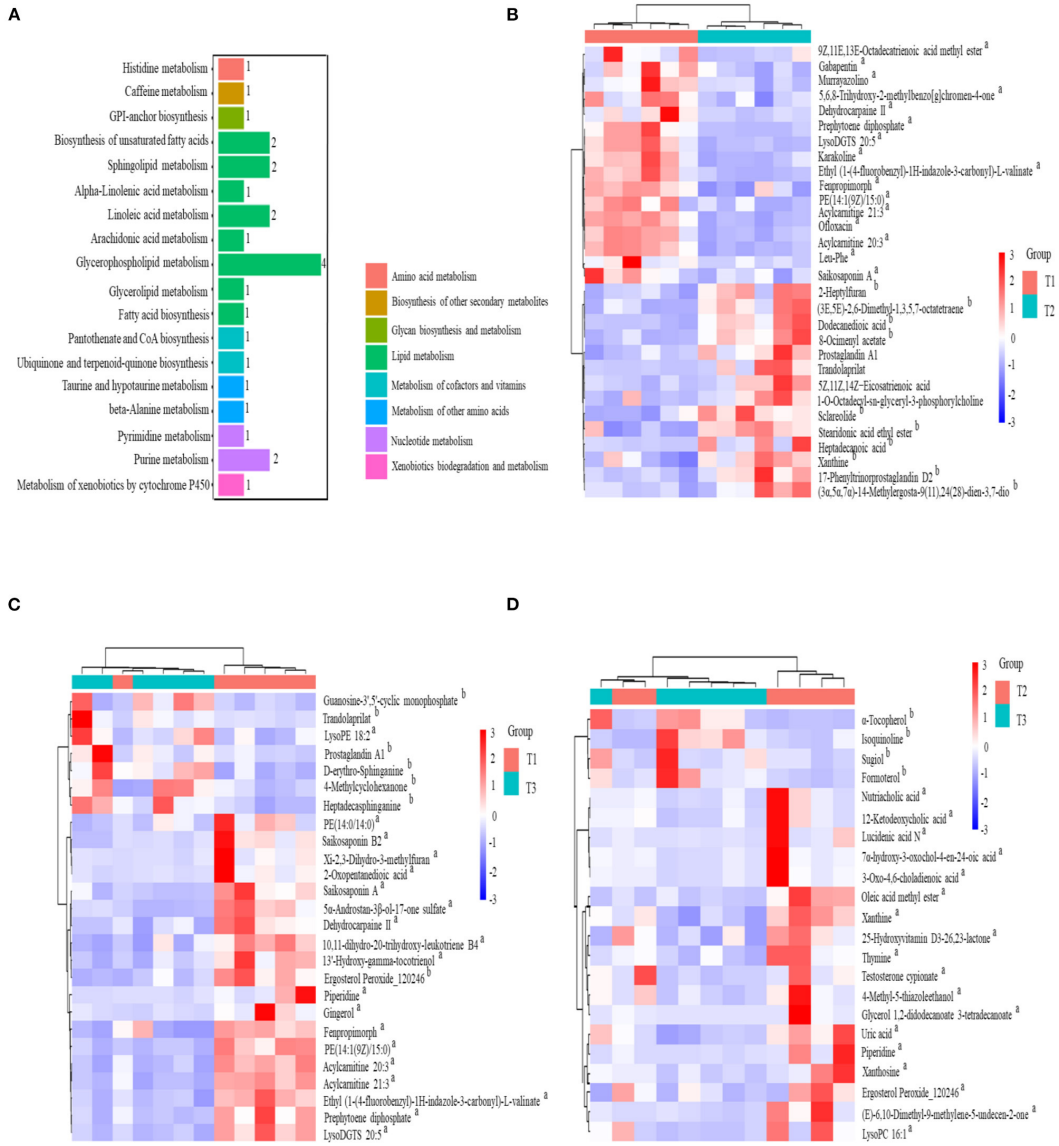
KEGG classification of differentially accumulated MS2 metabolite **(A)**. Heat-map of hierarchical clustering analysis for the dietary groups in cecal tissue of goats using the identified metabolites in positive and negative ionization mode **(B)** T1 vs. T2; **(C)** T1 vs. T3; **(D)** T2 vs. T3. The upregulated and downregulated metabolite denoted by superscript “a” and “b”, respectively.

The total extracts were subjected to UPLC-TQMS for non-targeted metabolomics to explore the effects of feeding different ratios of amylose/amylopectin on the metabolite profiles in the cecal tissue of weaned goats. The repeatability of each samples extract was assessed by an overlying analysis of the total ion current (TIC) in the quality control (QC) samples in negative and positive modes (Supplementary Figure 2). A partial least-squares discriminant analysis (PLS-DA) model exhibited that the three dietary groups were well-separated (Figure 8A). A total of 688, 619, and 286 peaks/metabolites were detected from the MS2 spectral data between the T1 vs. T2, T1 vs. T3, and T2 vs. T3 groups, respectively (Supplementary Table 1). All the metabolites were assigned in the KEGG database, and 34 KEGG pathways were influenced by the dietary ratios. Of the total, 48 differentially accumulated metabolites were assorted into “metabolism.” Of these, 14 and 3 metabolites were involved in lipid metabolism and nucleotide metabolism (Figure 9A). In total, 30, 26, and 22 differentially accumulated metabolites were identified in comparing the T1 vs. T2, T1 vs. T3, and T2 vs. T3 groups, respectively. Volcano plots were used to reveal differential metabolites, and 16 of 30 were upregulated while 14 of 30 were downregulated in the T2 group compared with the T1 group (Figure 8B). Compared with the T1 group, 19 of 26 metabolites were upregulated and 7 of 26 metabolites were down-regulated in the T3 group (Figure 8C). The accumulation patterns of differentially accumulated metabolites and QC samples were assessed using hierarchical cluster analysis (HCA) after normalizing the intensity value of each metabolite (Supplementary Figure 3). The HCA heat map constructed from DAMs revealed the difference in metabolites profiles of the dietary groups (Figures 9B–D).

## Discussion

The cecal microbes can ferment starch that escapes from the rumen and small intestinal digestion and produces SCFA, accounts for 12% of total SCFAs production in sheep due to ~17% cellulose degradation occurring in the cecum (30). A previous study has revealed that starch with increasing dietary amylose increases SCFA production and absorption (31). In the present study, increasing ratios of dietary amylose/amylopectin tended to improve acetate, propionate and valerate production in the cecum. This is consistent with the report of Tan and Zijlstra (32) who have shown that a diet consisting of high amylose ratio increases digesta acetate in the cecum and propionate and valerate in the colon. Feeding a high amylose diet did not affect the digesta butyrate concentration in the present study. A study has reported that the concentration of butyrate in the hindgut is affected by feeding a high amylose starch diet (33), while others indicate that a high percentage of amylose diet did not alter butyrate production (10). The discrepancy in butyrate production may result from a difference in the exposure period or could be the differences among individual animals. In this regard, the production of butyrate can be enhanced with time in the cecum of rats fed 90 g/kg of resistance potato starch for 0.5, 2, and 6 months (34). A similar response has also been reported in pigs-fed starch-containing different amylose proportions for 97 days (35), which may partially explain our results. Another possible explanation is that it is probably associated with increased uptakes of butyrate following increased production due to its uses as metabolic fuel for enterocytes.

It has been documented that high amylose resistant starch has protective effects against mucosa injury or inflammation and oxidative stress (36, 37). The protective effects of resistance starch were evidenced by lowering the levels of pro-inflammatory cytokines and TNF-α that would lead to a healthier immune status. In this sense, supplementation of 45 g/day of a high-amylose maize to pre-diabetes patients for 12 weeks resulted in significant decreases in the concentration of TNF-α. In the present study, the concentrations of IL-6 and TNF-α were decreased and IL-10 was increased by feeding a diet consisting of 50% high amylose, suggesting a potential to resist local inflammation in the cecal mucosa of weaned goats. This improvement in innate immunity matches the increased abundance of digesta-associated *Lactobacillus* and mucosa-associated *Akkermansia*, which has been linked to the protection from intestinal inflammation (38, 39).

To gain a profound picture of the effects of increasing ratio of amylose/amylopectin on the composition and dynamics of the mucosa-and-digesta associated cecal microbiota and their metabolite, we used a combination of in-depth sequencing of the 16S rRNA gene and metabolomics techniques. The results clearly showed that cecal bacterial composition was affected by feeding different ratios of dietary amylose/amylopectin. The predominant identified mucosa-and digesta-associated phyla were Firmicutes and Bacteroidetes, followed by the phyla Spirochaetes and Verrucomicrobia, which is in line with earlier reports on dairy goats fed different levels of rumen degradable starch (4). The presence of Spirochaetaes in the colon mucosa of lamb fed a non-pelleted and pelleted high-grain diet has been documented (40). Some species belongs to the phylum Spirochaetaes can degrade pectin (41) and xylan (42). Thus, the observed higher abundance of Spirochaetaes in the amylose/amylopectin ratio groups could be associated with the level of fiber in the diet. It has been widely reported that Proteobacteria consists of well-known pathogens such as *Escherichia coli* and *Salmonell* (43), while Verrucomicrobia consists of commensal bacteria such as *Akkermansia* (39). The present results showed that feeding a diet containing 50% high amylose decreased Proteobacteria and increased Verrucomicrobia abundances in cecal mucosa, showing that enrichments of commensal bacteria, which contributes to developing a healthier pattern of cecal environment.

A previous study has shown that supplementation of a diet containing a high proportion of amylose enriched the relative abundance of *Lactobacillus* (38) and *Faecalibacterium* (44). Some species of *Lactobacillus* can inhibit colonization of pathogens and lower the expression of TNF-α in a rat colitis model (45). Growing evidence suggests that some species from *Faecalibacterium* have a protective role through its anti-inflammatory potential (46). In the present study, the LEfSe analysis revealed that the abundances of digesta*-*associated *Lactobacillus* and *Faecalibacterium* were enriched at the expense of *Clostridium_sensu_stricto* and *Turicibacter* in goat-fed with a diet containing 50% high amylose. Similarly, enrichment of mucosa-associated *Akkermansia* at the cost of *Erysipelotrichaceae* and *Eimeria_praecox* was observed. The alterations of these species may be attributed to increasing SCFA production and the innate immune status. Combined with the comparable results in average daily gain and feed intakes between T2 and T3 groups (47), with the enrichments of beneficial bacteria, decreasing TNF-α, and increasing IL-10 in the T2 group, a 50% high amylose diet could have better outcomes compared with the other two diets.

The relative abundance of mucosa-associated *Roseburia, Lachnospiraceae_NK4A136_group and Marvinbryantia*, which belong to the *Lachnospiraceae* family, were increased by feeding a diet consisting of 100% high amylose. The enrichment of this family is associated with the improvement of fermentation and their specific products such as butyrate and propionate (48, 49). The concentration of butyrate in the 100% high amylose supplemented group was numerically higher even though statistically not differ among the groups. The abundance of mucosa-associated *Anaeroplasma* was enriched by feeding a diet with 100% high amylose. It has anti-inflammatory properties for the inhibition of chronic inflammation (50). In agreement with the previous study, a higher abundance of digesta-associated *Candidatus_Saccharimonas* in the present study could be associated with alterations of lipid metabolism (51, 52). In this sense, feeding a diet with a high amylose percentage has been shown to regulate fat metabolism and reduce fatty acids absorption in pigs (53).

Resistant starch may alter the cecal environment, thereby systemic exposure to some metabolites or other gut-derived factors that play a vital role in the biological ecosystem (54). In the present study, PLS-DA score plots revealed that variance in selected metabolites could discriminate among the three groups, suggesting that the ratios of amylose/amylopectin modulate the metabolic profiles of cecal tissue. This is in line with the previous study, which shows the metabolic profiles of hindguts can be altered by dietary starch (38). Resistance starch has been shown to affect the concentrations of metabolites that related to lipid metabolism or fatty acids such as arachidonic acid and hexadecanoic acid (8). Those dicarboxylic acids are obtained from the microbial hydrogenation of the non-conjugated dienoic acids built from linoleic acid (55) and could be produced by *Lactobacillus* species and *Eubacterium ventriosum* (56, 57). Compared with the T1 group, the levels of the majority of metabolites designated to the fatty acyls class, including dodecanedioic acid, heptadecanoic acid, stearidonic acid ethyl ester (5Z, 11Z, 14Z)-eicosatrienoic acid (arachidonic), and 8-Ocimenyl acetate, were downregulated in the T2 groups. In agreement with our report, feeding either resistance starch or maize grain diet decreases heptadecanoic and dodecanedioic acids levels in the rats and goats, respectively (7, 55). Conversely, the concentrations of those metabolites are higher in the feces of humans who consumed a diet containing resistant starch (58). Due to the central role of various fatty acids in host metabolism, higher absorption of those compounds by cecal tissue and the amount of starch reached to the cecum may be the possible reason for the observed lower concentration in the present study. Acylcarnitines are vital in regulating the equilibrium of intracellular sugar and lipid metabolism (59). They participate in various physiological activities such as fatty acids peroxidation (60) and uptakes of fatty acids into the inner membrane of mitochondria for β-oxidation to produce energy for cell activities (61). In our results, with an increasing ratio of amylose/amylopectin, the acylcarnitine levels were increased. These may have positive effects in maintaining cellular homeostasis and have specific roles in lipid metabolism. However, information about the gut microbes that facilitate the biotransformation of acylcarnitines is not yet clarified (62).

The metabolome result showed that the levels of ofloxacin and saikosaponins were upregulated in response to increase the dietary amylose/amylopectin ratio. Both of them are likely derived from diets and cannot be synthesized by gut microbes. Ofloxacin is a quinolone carboxylic acid derivative and has been reported to have antibacterial and pharmacokinetic properties in a different state of disease (63, 64), while saikosaponins have shown a protective role against a wide variety of age-related diseases (65). Increasing the concentration of those metabolites could attribute to improve the health status of goats due to the central modulatory effects of those metabolites. It has been documented that 12-Ketodeoxycholic and 3-Oxo-4, 6-choladienoic acids are the secondary bile acid produced by intestinal bacteria (66). *Clostridium* and *Ruminococcus* species can produce secondary bile acids (67), which have been shown to involve in several physiological process, including antimicrobial peptides production. In the present study increasing in concentrations of the secondary bile acid metabolites may indicate a high rate of deconjugation of bile salts and alters the microbial metabolism in the cecum, possibly due to an increase in starch flow to the intestine. Another metabolite significantly reduced by a ratio of amylose/amylopectin was alpha-Tocopherol, which cannot be produced by gut microbes and possibly arose from the diet (68). The observed decreased level of α-tocopherol in the T3 group compared with the T2 group could be due to increased uptake of α-tocopherol as the ratio of amylose increased. This metabolite has several biological functions, including scavenging the lipid peroxyl radicals, regulation of cell growth, and cell signaling functions (69, 70).

The other metabolite increased by a ratio of amylose/amylopectin was piperidine, which is produced by gut microbes and has an adverse influence on the host (71). The concentrations of several metabolites were increased by the ratio of amylose/amylopectin (Figure 9). Of them, some can serve as a biomarker of health benefits. For example, Lyso-diacylglyceryltrimethylhomoserine (LysoDGTS) is associated with a reduction of cellular lipid accumulation (72). In addition, phosphatidylethanolamines (PE) have been shown linkage with the relative abundances of *Faecalibacterium* (73), which is consistent with the present results that indicated enrichment of *Faecalibacterium* and PE in the T2 group. Members of the PE reported to have a structural role in biological membranes and cell division and could use as a key biomarker for cardiovascular disease (74). However, the functional role of these metabolites in the goat cecal tissue requires further investigation. Overall, lower concentrations of some metabolites that have a crucial role in the health benefits of animals are not an ideal outcome regardless of potential benefits gained when feeding a diet containing a high amylose ratio.

## Conclusion

The microbiome and metabolomics results showed that a high ratio of amylose/amylopectin modulates the digesta-and mucosa-associated cecal microbial community and metabolic profiles more likely toward a healthier pattern and host-friendly cecal environment. Feeding a diet containing 50% high amylose had better outcomes, as characterized by the enrichments of commensal bacteria such as *Akkermansia, Lactobacillus*, and *Faecalibacterium*, increased production of valerate, and decreased production of pro-inflammatory cytokines. The metabolomics results revealed the modification of the metabolic signatures of cecal tissue, evidenced by up- and -downregulation of several metabolites such as acylcarnitines and α-Tocopherol. These findings provide new insights into how the ratios of amylose/amylopectin modulate microbial fermentation, lead to proliferation of commensal microbiota and metabolites and thereby improve the cecal environment of weaned goats.

## Data Availability Statement

The datasets presented in this study can be found in online repositories. The names of the repository/repositories and accession number(s) can be found at: https://www.ncbi.nlm.nih.gov/bioproject/PRJNA759377.

## Ethics Statement

The animal study was reviewed and all experimental animal procedures were performed following the protocols approved by the Animal Care and Use Committee of the Institute of Subtropical Agriculture, The Chinese Academy of Sciences, Changsha, China.

## Author Contributions

KG: conceptualization, data analysis, and writing—original draft. KC: investigation and data acquisition. TW and MA: data analysis, review, and editing. JH, WJ, and WS: resource, review, and editing. ZH and ZT: conceptualization, project administration, supervision, review, and editing. All authors have read and approved the final manuscript.

## Funding

This work was supported by National Natural Science Foundation of China (31730092), Strategic Priority Research Program (Grant Nos. XDA26040304 and XDA26050102), STS Project of the Chinese Academy of Sciences (KFJ—STS-ZDTP−075), Hunan Key Research and Development Program (2020NK2049), and Innovation Province Project (2019RS3021).

## Conflict of Interest

The authors declare that the research was conducted in the absence of any commercial or financial relationships that could be construed as a potential conflict of interest.

## Publisher's Note

All claims expressed in this article are solely those of the authors and do not necessarily represent those of their affiliated organizations, or those of the publisher, the editors and the reviewers. Any product that may be evaluated in this article, or claim that may be made by its manufacturer, is not guaranteed or endorsed by the publisher.
