# Supplementary material for: Dietary Amylose/Amylopectin Ratio Modulates Cecal Microbiota and Metabolites in Weaned Goats
Source: Front Nutr. 2021 Nov 23;8:774766. doi: 10.3389/fnut.2021.774766 (PMC8697430; doi:10.3389/fnut.2021.774766)
Supplement: Supplementary file 1 [file Data_Sheet_1.docx]

SUPPLEMENTARY FIGURES

A

B


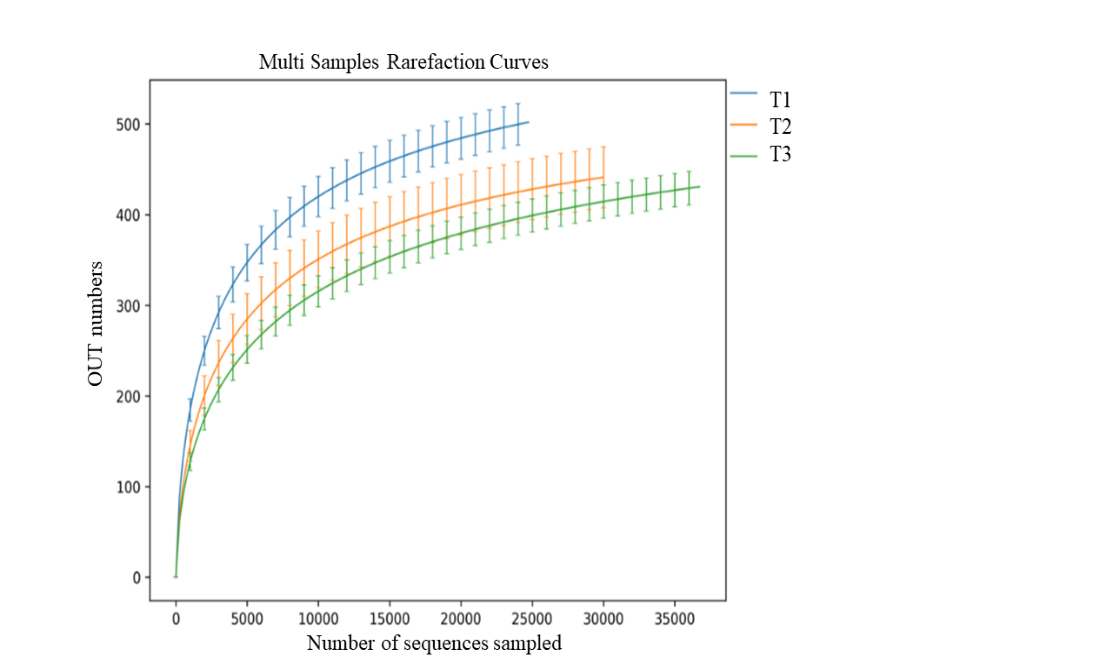

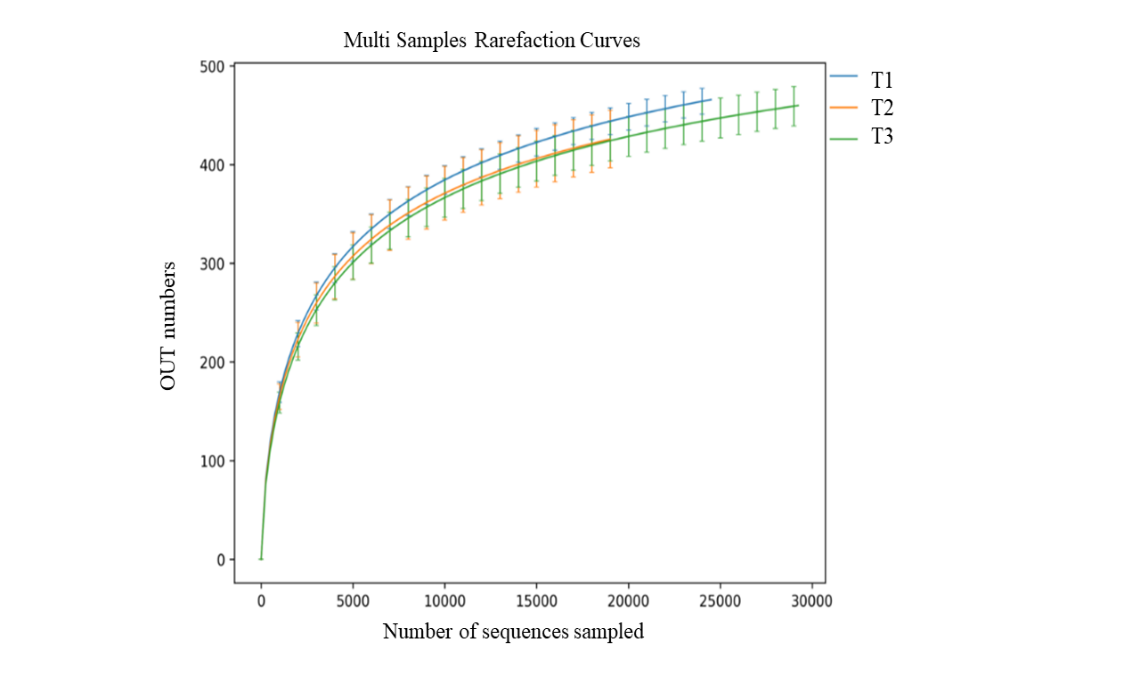


Figure 1: Rarefaction curves for cecal tissue (A) and digesta samples (B). T1 (normal corn 100%, high amylose corn 0%); T2 (normal corn 50%, high amylose corn 50%); T3 (normal corn 0%, high amylose corn 100%).


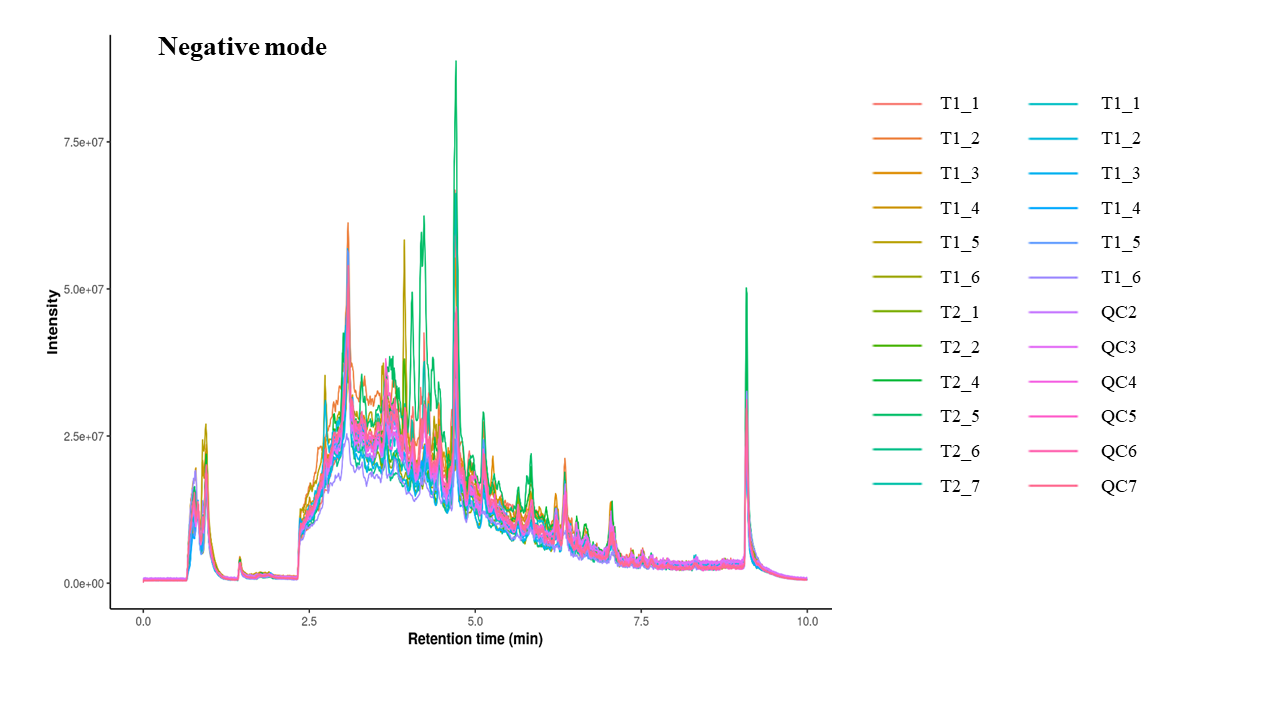


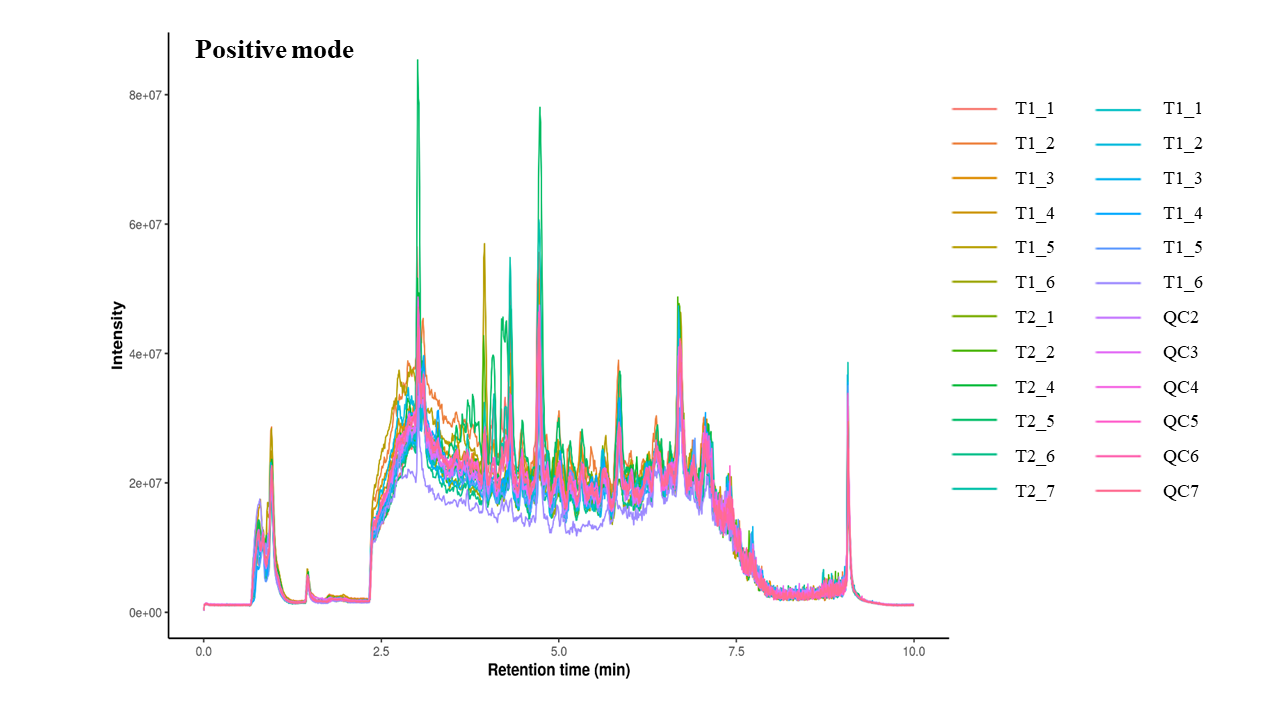


**Figure S2**. Overlapping analysis of the total ion current (TIC) in different quality control (QC) samples.


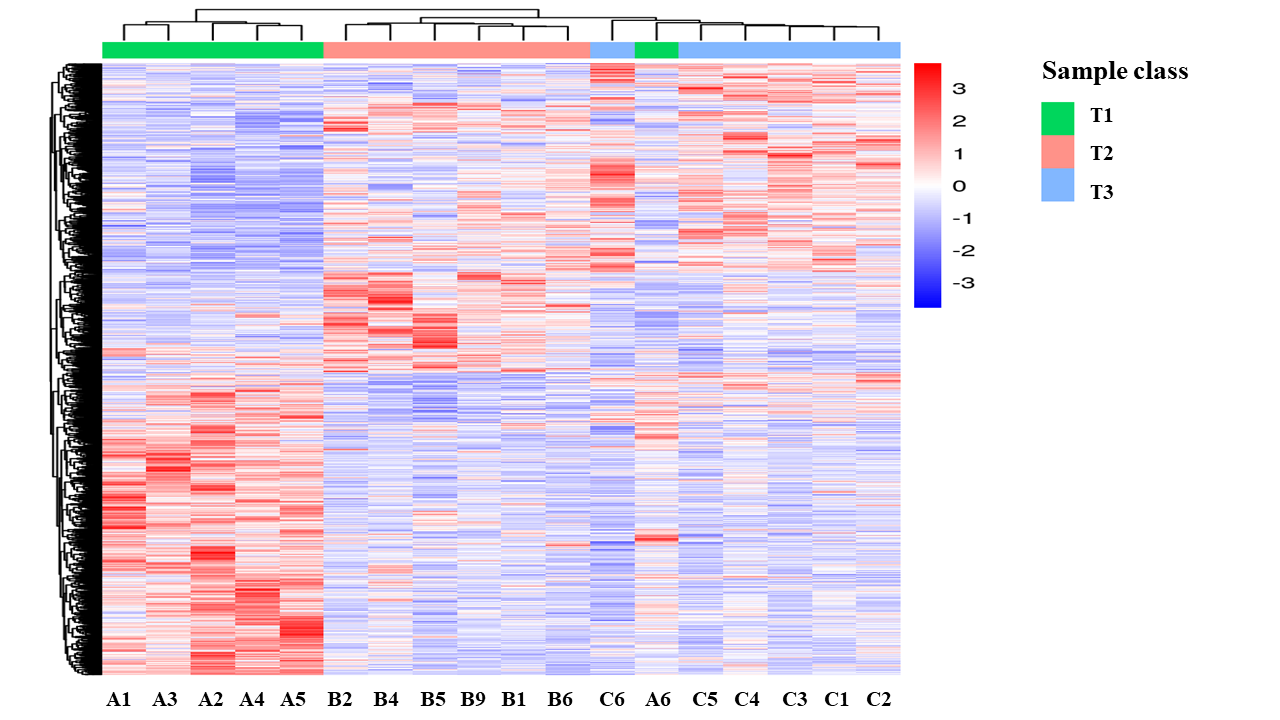


**Figure S3**. Heat map representing the hierarchical cluster analysis in differential accumulated metabolites and QC samples.
